# Filamin A interacting protein 1-like (FILIP1L) has mitochondrial localization

**DOI:** 10.17912/micropub.biology.001572

**Published:** 2025-03-25

**Authors:** Sarah Henretta, Karisa Bockley, Jacob Odell, Jan Lammerding

**Affiliations:** 1 Meinig School of Biomedical Engineering, Cornell University, Ithaca, NY 14853; 2 Weill Institute for Cell and Molecular Biology, Cornell University, Ithaca, NY 14853; 3 Graduate Field of Biochemistry, Molecular and Cell Biology, Cornell University, Ithaca, NY 14853

## Abstract

Filamin A interacting protein 1-like (FILIP1L) is a multifunctional protein that plays a role in cancer progression, apoptosis, and angiogenesis. However, FILIP1L remains a relatively underexplored protein, and many of FILIP1L’s key mechanisms and interacting partners are still unknown. Using immunofluorescence staining for endogenous FILIP1L and high-resolution confocal microscopy, we show that FILIP1L colocalizes with the mitochondrial marker, TOM20, suggesting a novel connection between FILIP1L and mitochondria. Further exploring the relationship between FILIP1L and mitochondria might provide a deeper understanding of the emerging function of FILIP1L in health and disease.

**Figure 1. FILIP1L exhibits mitochondrial localization in several human cell lines:**
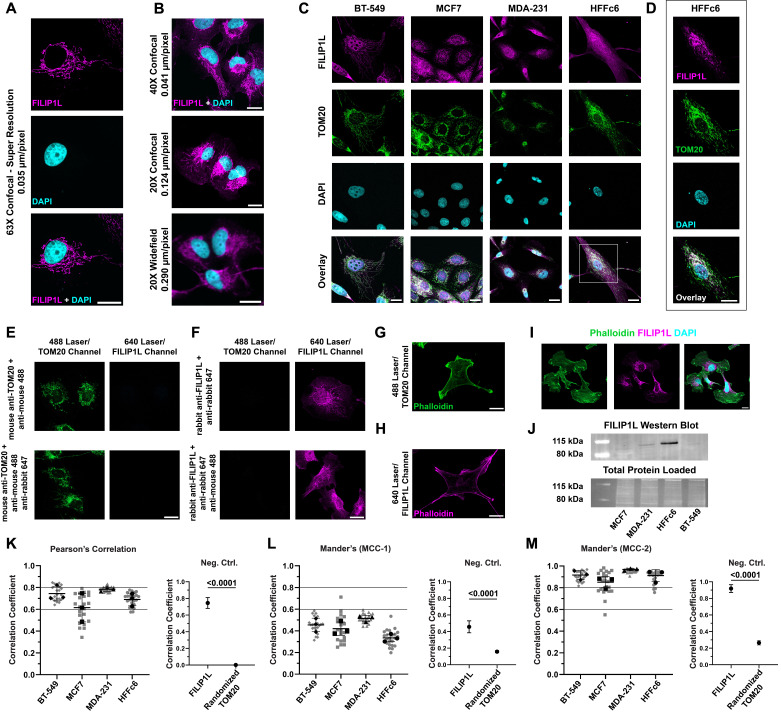
( **A** ) The FILIP1L subcellular localization strongly resembles mitochondrial distribution when imaged by confocal and super resolution microscopy. Scale bars = 20 µm. ( **B** ) The mitochondrial localization is less apparent when imaged by widefield microscopy at lower magnifications and lower image resolution. Scale bars = 20 µm. ( **C** ) FILIP1L colocalizes with the mitochondrial marker TOM20 in four human cell lines. The colocalization is less obvious in the HFFc6 cells but still visible. Brightness levels were linearly adjusted for each image to enhance the visualization of FILIP1L and TOM20. ( **D** ) HFFc6 colocalization is more readily apparent when the image contrast and minimum pixel value are increased to reduce diffuse signal in the cytoplasm. ( **E, F** ) Immunofluorescence labeling of BT-549 cells to address potential bleed-through of secondary antibody or non-specific recognition of primary antibody. Cells were labeled with either the mouse primary antibody against TOM20 (E) or rabbit primary antibody against FILIP1L (F), and fluorescently conjugated secondary antibodies against either one (top) or both primary antibodies (bottom). The anti-mouse secondary antibody is visible in the green channel (488 nm excitation) and the anti-rabbit secondary antibody in the magenta channel (647 nm excitation). ( **G, H** ) Phalloidin staining of BT-549 cells in the 488 and 647 channels, without any antibody labeling against FILIP1L, failed to show any mitochondrial localization patterns similar to the FILIP1L and TOM20 patterns, ruling out mitochondrial autofluorescence as an explanation. ( **I** ) Moreover, co-staining BT-549 cells with FILIP1L and Phalloidin revealed that the FILIP1L fluorescent signal when excited with a 640 nm laser is distinct from excitation with the 488 nm laser. ( **J** ) Western blot analysis identified prominent bands near the expected molecular weights for FILIP1L’s two largest isoforms (~100 kDa). ( **K** ) Pearson’s Correlation Coefficients for FILIP1L and TOM20 fluorescence signals for four human cell lines indicate a strong correlation between the two proteins. ( **K,** right) FILIP1L and TOM20 have significantly higher Pearson’s Correlation Coefficient values than FILIP1L to the corresponding TOM20 image with its pixels randomized. ( **L-M** ) MCC-1 and MCC-2 values suggest moderate and strong colocalization, respectively, between the two proteins. ( **L-M,** right) In BT-549 cells, FILIP1L and TOM20 have significantly higher MCC-1 and MCC-2 values than FILIP1L to the corresponding TOM20 image with its pixels randomized. Grey data points in each graph represent one image taken, including multiple cells, whereas black data points represent the means of each replicate. Black lines across all graphs indicate strong (y=0.6, lower line) and extremely strong (y=0.8, upper line) correlation thresholds. Scale bars = 20 µm.

## Description

Filamin A interacting protein 1-like (FILIP1L) is a multifunctional protein with five transcription variants (Kwon & Libutti, 2014; Jing et al., 2021). FILIP1L is a tumor suppressor-like protein that plays a role in apoptosis, angiogenesis, and cancer progression (Kwon et al., 2008, 2016; Jing et al., 2021) and that is downregulated in multiple cancers, including ovarian, breast, colon, lung, and pancreatic cancer, possibly due to DNA methylation of the FILIP1L promoter (Kwon et al., 2013). FILIP1L expression is inversely correlated with the invasive potential in these cancers (Kwon et al., 2008, 2013; Kwon & Libutti, 2014; Kwon et al., 2016) and FILIP1L overexpression can inhibit cell migration and invasion (Kwon et al., 2008). Additionally, FILIP1L is implicated in the Wnt signaling pathway through its promotion of β-catenin degradation and suppression of epithelial-mesenchymal transition (Kwon et al., 2016).

As we investigated FILIP1L protein expression and localization in multiple human breast cancer cell lines, we identified a distinct FILIP1L subcellular localization pattern resembling that of mitochondria ( **Fig. 1A** ). FILIP1L has been previously reported to localize to the cytoplasm (Kwon et al., 2008; Kwon & Libutti, 2014), consistent with what we observed when imaging cells at low magnification using widefield microscopy ( **Fig. 1B** ). In contrast, the mitochondrial localization pattern was only apparent using high-magnification immersion objectives (i.e., 40×-water and 63×-oil) in combination with high-resolution (AiryScan) confocal microscopy. This difference in the image acquisition settings might explain why FILIP1L mitochondrial localization was not observed previously (Hu & Mivechi, 2011).

To confirm that FILIP1L localization matched that of mitochondria, we co-stained three human breast cancer cells (BT-549, MCF7, MDA-231) with a polyclonal antibody against endogenous FILIP1L and an antibody against TOM20, a common mitochondrial marker (Eliyahu et al., 2010). These experiments revealed a strong overlap between FILIP1L and TOM20 ( **Fig. 1C** ). All three human breast cancer cell lines exhibited FILIP1L localization patterns resembling mitochondrial distribution based on the strong colocalization between FILIP1L and TOM20. To assess whether this pattern was specific to female breast cancer cells or more broadly applicable, we included human foreskin fibroblasts (HFFc6) to our panel of cell lines. The non-cancerous HFFc6 cells exhibited a similar mitochondrial distribution of FILIP1L as the breast cancer cell lines. FILIP1L exhibited some inter- and intra-cell line heterogeneity, localizing not only to the mitochondria, but also diffusely throughout the cytoplasm, to the nucleus, or in combination of these patterns ( **Fig. 1C** ). Nonetheless, we identified a consistent colocalization between FILIP1L and mitochondrial localization patterns, including in the HFFc6 cells, which displayed the most diffuse cytoplasmic FILIP1L expression. The mitochondrial localization in the HFFc6 cells became even more apparent when enhancing the image contrast ( **Fig. 1D** ).

We performed several control experiments to rule out alternative explanations for the mitochondrial localization of the FILIP1L immunofluorescence labeling. Staining with individual antibodies against TOM20 ( **Fig. 1E** ) and FILIP1L ( **Fig. 1F** ), with either one or multiple secondary antibodies, demonstrated that the mitochondrial localization of the FILIP1L signal did not arise from bleed-through or non-specific secondary antibody recognition **.** Furthermore, to rule out autofluorescence or other signal causes, we imaged fixed BT-549 cells without any FILIP1L or TOM20 labeling, using Phalloidin as a cytoplasmic marker. In these experiments, we did not observe any subcellular localization patterns similar to the TOM20 and FILIP1L pattern ( **Fig. 1G, H** ). Finally, we co-stained for FILIP1L and Phalloidin in BT-549 cells together to confirm that FILIP1L specifically associates with TOM20 rather than broadly interacting with the actin cytoskeleton ( **Fig. 1I** ) **.** To verify that the anti-FILIP1L antibody used in our experiments recognizes endogenous FILIP1L, we probed FILIIP1L expression in the four cell lines by western blot analysis and for each cell line observed one or two bands at around the molecular weights predicted for FILIP1L and in line with antibody testing data from the manufacturer ( **Fig. 1J** ).The immunogen used to generate this antibody (aa 566-661 from human FILIP1L) is present in all FILIP1L isoforms except isoform 4. Based on the immunogen sequence and the molecular weights of the observed bands, we expect that the antibody will detect the major isoforms 1 and 2, matching the observed molecular weights. We did not detect prominent non-specific bands at other molecular weights, suggesting that the antibody is indeed specific for FILIP1L.

To quantify the degree of spatial correlation between FILIP1L and TOM20, we calculated the Pearson’s Correlation Coefficient and Mander’s Correlation Coefficients. The Pearson’s Correlation Coefficient evaluates the linear correlation between the fluorescence intensities of two signals across corresponding pixels in an image, providing an overall measure of spatial association (Bolte & Cordelières, 2006). The mean Pearson’s Correlation Coefficient values across all cell lines indicate a strong positive correlation between FILIP1L and TOM20, with Pearson’s Correlation Coefficient values of 0.6 or higher ( **Fig. 1K** ) **.** The Mander’s Correlation Coefficients quantifies the extent of overlap between two signals, offering a direct measure of colocalization. In our analysis, Mander’s Correlation Coefficient-1 (MCC-1) represents the fraction of FILIP1L’s signal overlapping with TOM20, and Mander’s Correlation Coefficient-2 (MCC-2) represents the fraction of TOM20’s signal overlapping with FILIP1L. The MCC-1 values, which ranged from 0.36 in HFFc6 cells to 0.51 in MDA-231 cells, did not demonstrate a strong correlation ( **Fig. 1L** ), likely because FILIP1L exhibited more diffuse subcellular localization across the cytoplasm and nucleus, in addition to its mitochondrial localization, whereas TOM20 was localized to very specific locations throughout the cytoplasm. The MCC-2 values support this interpretation, as they were consistently higher than the MCC-1 values, ranging from 0.85 in the MCF7 cells to 0.97 in the MDA-231 cells, indicating that a large fraction of the TOM20 fluorescence signal overlaps with the FILIP1L signal ( **Fig. 1M** ). Together, the Pearson’s Correlation Coefficient and Mander’s Correlation Coefficients provide a comprehensive assessment of both the linear relationship between fluorescence signals (Pearson’s Correlation Coefficient) and the degree of physical overlap (Mander’s Correlation Coefficients) for the two proteins. Our results reveal a high degree of overlap between FILIP1L and TOM20 across the entire cell panel, as the consistently high Pearson’s Correlation Coefficients and MCC-2 values suggest a strong correlation between FILIP1L and TOM20 in terms of both intensity and spatial localization. To assess the significance of the correlation coefficients, we compared the BT-549 correlation coefficients with those calculated between FILIP1L images and their corresponding TOM20 image with randomized pixels ( **Fig. 1K- M,** right). All three correlation coefficients were statistically distinct from the randomized controls, confirming that the observed colocalization is significant. To minimize bias of the image background when randomizing TOM20 pixels, we used cropped FILIP1L and TOM20 images containing only regions covered entirely with cells. This approach ensured that the randomization process did not include any cell-free background pixels, which could artificially reduce colocalization since the FILIP1L and TOM20 signal would not localize outside of cell-covered regions.

We acknowledge several limitations of the current study. The exact reason for FILIP1L’s colocalization with mitochondria remains unclear and additional studies are needed to confirm the biological relevance of our findings. Our study uses only a single, albeit commonly used, polyclonal antibody. Further testing with additional antibodies would be needed to determine whether our findings are antibody specific, isoform specific, or more broadly applicable. Moreover, repeating this study in cells with a fluorescently tagged FILIP1L protein could remove some of the uncertainty introduced by using immunofluorescence labeling with antibodies, but would conversely raise potential questions about the effect of the fluorescent tag and effects of FILIP1L overexpression above the endogenous background. Finally, we show consistent signal correlation and spatial overlap between FILIP1L and TOM20, but additional experiments, such as co-immunoprecipitation or proximity ligation assays, are needed to determine if FILIP1L interacts directly with mitochondria. Future studies should focus on identifying the binding partners of FILIP1L and its interaction with mitochondrial elements.

Nonetheless, based on our findings using multiple lines of evidence and extensive experimental controls, we report that FILIP1L has a previously non-recognized mitochondrial localization. Our findings suggest that FILIP1L might interact with mitochondrial proteins, regulate mitochondrial dynamics, and/or binds to the mitochondrial membrane to influence cellular signaling and function. Further research could uncover novel mechanisms connecting FILIP1L to mitochondrial function, with consequences in normal biological functions and pathological processes.

## Methods

***Cell culture.*** BT-549 cells were cultured in in RPMI 1640 Medium supplemented with 10% Fetal Bovine Serum (FBS) + 1% Penicillin-Streptomycin. MDA-231 and MCF7 cells were maintained in Dulbecco's Modified Eagle Medium (DMEM) supplemented with 10% FBS + 1% Penicillin-Streptomycin. HFFc6 cells were cultured in DMEM supplemented with 20% FBS + 1% Penicillin-Streptomycin. All cells were grown at 37°C and with 5% CO _2_ .

***Immunofluorescence labeling and imaging.*** The day before fixing, 30,000 cells were seeded on glass coverslips coated with fibronectin (1:200 in 1× dPBS) and incubated at 37°C and 5% CO _2_ overnight. The following morning, cells were fixed in prewarmed 4% paraformaldehyde, washed with PBS, permeabilized using PBS + 0.1% Triton-100X for 10 mins at room temperature (RT), and blocked with blocking solution (PBS + 0.2% Triton-100X, 0.05% Tween20, and 3% BSA) for 1 h at RT. Cells were stained with primary antibodies diluted 1:100 in blocking solution and incubated at 4°C overnight. Afterwards, the cells were washed with PBS, incubated with secondary antibodies and DAPI for 1 h at RT, mounted on glass slides using Hydromount, and dried in the dark at RT. Representative confocal images were acquired on a Zeiss LSM 900 microscope with AiryScan module using a 20× (NA 0.80), 40× water immersion (NA 1.20), or 63× oil immersion (NA 1.40) objectives. For colocalization analysis, z-stacks were taken on the Zeiss LSM 900 microscope with AiryScan module using the 40× water immersion objective. Z-stacks of each fluorescent channel were acquired sequentially using the 405 nm, 488 nm, and 640 nm laser lines and capturing a broad fluorescence emissions spectrum within the 400 nm to 700 nm range. Representative widefield fluorescence images were acquired on a Keyence BZ-X810 with a 20× objective (NA 0.75).

***Immunoblotting.*** Approximately 4×10 ^5^ cells for each cell line were lysed on ice for 2 mins in high salt RIPA buffer containing 12 mM sodium deoxycholate, 50 mM Tris-HCl pH 8.0, 750 mM NaCl, 1% (v/v) NP-40 alternative and 0.1% (v/v) SDS in ultrapure water. Lysates were vortexed for 5 min, sonicated (Branson 450 Digital Sonifier) for 30 s at 36% amplitude, boiled for 2 min, centrifuged at 4°C for 10 min at 14,000 g and stored at −70°C. Equal protein amounts were denatured in 5× Laemmli buffer by boiling for 3 min, loaded onto 4–12% Bis-Tris gels (Invitrogen NP0322), separated for 1.5 h at 100 V, then transferred for 1 h at 16 V onto PVDF membrane. Membranes were blocked for 1 h in blocking buffer containing 3% BSA in Tris-buffered saline plus 1% Tween 20. Rabbit anti-FILIP1L was added 1:1000 in blocking buffer overnight. Secondary antibodies were added for 1 h at room temperature in blocking buffer, followed by three 10-min washes. Membranes were imaged using the Odyssey Licor scanner and then cropped and brightness and contrast was adjusted using Image Studio Lite (version 5.2) software.

***Colocalization analysis.*** To analyze the images, maximum-intensity projections were generated of each z-stack. Pearson’s and Mander’s Correlation Coefficients between the 488 nm (TOM20) and 647 nm (FILIP1L) excitation channels were calculated using the JACoP plugin on ImageJ (Bolte & Cordelières, 2006). Images were thresholded before calculating the Mander’s coefficients to eliminate the bias from the largely black background of the images. The MCC-1 value represents the fraction of the signal from 640 nm laser excitation that overlaps the 488 nm laser excitation, and the MCC-2 value represents the fraction of the signal from 488 nm laser excitation that overlaps the 640 nm laser excitation.

***Randomized images*** . A max intensity projection of FILIP1L and TOM20 expression in BT-549 cells was cropped in multiple regions to retain only cellular portions, ensuring no unoccupied space remained. The pixels in the TOM20 images were randomly shuffled using a custom ImageJ macro to randomize TOM20 signal distribution. Colocalization coefficients were calculated between the FILIP1L and randomized TOM20 images as previously described.

***Statistical analysis*** . Unpaired *t* -tests were performed using GraphPad Prism to assess the significance between the FILIP1L-TOM20 coefficients and FILIP1L-randomized TOM20 coefficients in BT-549 cells. Statistical comparisons across cell lines were not conducted, as they were beyond the scope of this study.

## Reagents

**Table:** 

**Cell Type**	**Species**	**Source**
MDA-231	Human	ATCC [HTB-26]
BT-549	Human	ATCC [HTB-122]
MCF7	Human	ATCC [HTB-22]
HFFc6	Human	These cells were a generous gift from Rachel McCord (U Tennessee) and Job Dekker (HHMI, University of Massachusetts Chan Medical School)

**Table:** 

**Primary Antibody**	**Host Species**	**Source**
FILIP1L	Rabbit	Proteintech, 30134-1-AP
TOM20	Mouse	Santa Cruz, sc-17764

**Table:** 

**Secondary Antibody**	**Target Species**	**Source**
Donkey anti-Rabbit IgG Alexa Fluor 647	Rabbit	Invitrogen, A-31573
Donkey anti-Mouse IgG Alexa Fluor 488	Mouse	Invitrogen, A-21202
IRDye 800CW Donkey anti-Rabbit IgG	Rabbit	Licor, 926-32213

**Table:** 

**Reagent**	**Source**
Dulbecco's Modified Eagle Medium (DMEM)	Life Technologies, 11965118
RPMI 1640 Medium	Life Technologies, 11875093
Fetal Bovine Serum (FBS)	VWR, MP130050
Penicillin-Streptomycin	Life Technologies, 15070063
10X dPBS	Life Technologies, 14200075
0.25% Trypsin-EDTA	Fisher Scientific, 25200056
Fibronectin Human Purified	Krackeler Scientific, 45-FC010
Tween20	Millipore Sigma, 9005-64-5
Bovine Serum Albumin	VWR, AAJ65788-22
Triton X-100	Millipore Sigma, 9036-19-5
16% Paraformaldehyde	Life Technologies, 043368.9M
DAPI	Life Technologies, 62248
Hydromount	Electron Microscopy Sciences, 17966
